# Rabies Prophylaxis for Pregnant Women

**DOI:** 10.3201/eid1312.070157

**Published:** 2007-12

**Authors:** Mohamed E. Abazeed, Sandro Cinti

**Affiliations:** * University of Michigan Medical School, Ann Arbor, Michigan, USA; † University of Michigan Hospitals/Ann Arbor VA Medical Center, Ann Arbor, Michigan, USA

**Keywords:** Bat, undetectable bite exposure, immune globulin, vaccine, letter

## Abstract

Rabies Prophylaxis for Women

**To the Editor:** Rabies poses a 100% risk for death to pregnant women and an indeterminate risk to the fetus (*1*,*2*). Although a theoretical risk exists for adverse effects from rabies immune globulin and killed rabies virus vaccines, several studies assessing the safety of this treatment have failed to identify these risks (*3*–*6*). Indeed, the consensus is that pregnancy is not a contraindication to rabies postexposure prophylaxis (PEP) (*7*). Despite this concensus, healthcare providers resist treating pregnant women with rabies PEP. We describe a case of a pregnant woman with uncertain rabies exposure.

A 35-year-old pregnant woman (at 34 weeks gestation) sought treatment 3 weeks after being exposed to a bat. The patient reported awakening at 3:00 am to find a bat flying in her bedroom. She attempted to confine the bat to 1 section of the home and then called for help. A relative trapped and retrieved the bat, then disposed of the animal without further incident. The patient denied being bitten by the bat, and she had no obvious bite marks after the event. Initially, the patient sought information from online resources, her primary care physician, and her obstetrician. She was uncertain whether rabies PEP was warranted, given what she believed to be the low probability of the bat being rabid and the low likelihood of her having had direct exposure to the bat. The patient did express concern about the safety of rabies PEP in pregnant women. Because no unequivocal recommendations were made by either her primary care physician or obstetrician, she sought further advice from the Infectious Diseases Department at the University of Michigan on how best to proceed.

The 1999 recommendations of Centers for Disease Control and Prevention Advisory Committee on Immunization Practices state, “... postexposure prophylaxis can be considered for persons who were in the same room as the bat and who might be unaware that a bite or direct contact had occurred ...” (*8*). Bat bites may not be apparent when they occur, even with careful examination. In fact, most of the recent human rabies patients have no known history of exposure to a rabid animal (*9*,*10*). Of the 21 cases of bat-associated rabies in the United States during 1980–1999, 12 (57%) occurred in persons with apparent bat contact but no detectable bites (*8*). Our patient woke up with a bat flying in her room and did not know how long it had been there. The best course of action would have been to test the bat for rabies. However, because the animal had already been disposed of, laboratory testing for rabies was not possible. Furthermore, given that 5%–9% of bats tested in Washtenaw County, Michigan, are positive for rabies (www.mdch.state.mi.us/pha/epi/cded/cd/batcoframe.htm), the exposure risk was not insignificant. Therefore, it was our opinion that this patient qualified for rabies PEP.

Several studies of the safety of rabies PEP for pregnant patients demonstrated no association between treatment and adverse outcomes (*3*–*6*). In 1 study, tissue culture-derived vaccines and human immune globulin did not lead to an increased risk for congenital anomalies; no effects were observed on intrauterine or infant growth or development with a follow-up period of 1 year postpartum (*6*). Although these studies are not comprehensive in their assessment of all reproductive outcomes, they do suggest that PEP is generally safe.

On the basis of the exposure and our literature review, we recommended that the patient receive rabies PEP. After discussing options with her husband, the patient chose not to receive treatment, citing continued concern about the effect of rabies PEP on the fetus. There must be a greater public health effort to educate clinicians and the public about proper response to bat exposures, particularly undetectable bite exposures such as this case. Had public health authorities been contacted to collect and test the captured bat for rabies, there would have been no ambiguity as to the appropriate course of action.
